# Learning professionalism through hidden curriculum: Iranian medical students’ perspective

**Published:** 2018-09-09

**Authors:** Sajjad Azmand, Sedigheh Ebrahimi, Mohammadtaghi Iman, Omid Asemani

**Affiliations:** 1 *PhD Candidate of Medical Ethics, Department of Medical Ethics, Shiraz University of Medical Sciences, Shiraz, Iran. *; 2 *Associate Professor, Department of Medical Ethics, Shiraz University of Medical Sciences, Shiraz, Iran.*; 3 *Professor, Department of Sociology, Shiraz University, Shiraz, Iran.*; 4 *Assistant Professor, Department of Medical Ethics, Shiraz University of Medical Sciences, Shiraz, Iran.*

**Keywords:** *Hidden curriculum*, *Professionalism*, *Undergraduate medical student*, *Content analysis*, *In-depth interview*

## Abstract

Learning professionalism is a central topic in medical education. While many factors could affect the educational process of professionalism, hidden curriculum is considered one of the most important ones. As the working components of a hidden curriculum might be specific to the settings, this study explored its components in terms of professionalism and ethical conduct from the viewpoint of Iranian undergraduate medical trainees.

Semi-structured and in-depth interviews were used to collect medical students' experiences and viewpoints, which were then analyzed through simple content analysis and the codes and categories were extracted. Finally, themes were derived as the central organizing concepts.

Saturation occurred after 17 interviews. Seven main themes were extracted as the working components of hidden curriculum regarding professionalism in the setting: ‘convenient patients’, ‘evaluate me’, ‘trust as the base of team interactions’, ‘perceiving encouragement’, ‘relationship satisfaction and authenticity’, ‘workload and students’ well-being’ and ‘role modeling at the heart of professionalism’.

Students' perception and experiences are a rich source of gaining a deeper understanding of the working hidden curriculum. In this study, two groups of human-related and environment-related elements were extracted. They were effective in the formation of the current 'ethical climate', which shaped the professional and ethical identity of medical trainees. Moreover, specific plans regarding the condition of the settings may provide opportunities for medical educators to enhance professionalism in their institutions.

## Introduction

There is still a noticeable gap between what medical educators intend to teach medical trainees through formal curriculums and what they learn through non-formal or hidden curriculums (1). The existing literature shows that teaching, learning and evaluating professionalism have been a matter of serious concern for medical educators (2 - 4). However, they worry about how medical students’ professional identity, attitudes and behaviors are formed and developed according to formal plans (5). Educators know that designing professional guidelines and instructions, developing assessment tools, making curriculum reforms, etc. are helpful but not enough, and the working hidden curriculum should also be taken into account (6, 7). Therefore, enhancing professionalism through formal reforms without considering specific hidden curriculums and their components in educational contexts is doomed to fail (8).

Haffetry and Frank introduced the concept of hidden curriculum in medical education in their article “The hidden curriculum, ethics teaching, and the structure of medical education” (9). This concept opens different arenas to medical educators and makes them think how hidden curriculum might operate in the shadow of formal curriculum (10). However, there is no consensus about its definition, nature and working process in medical contexts (11, 12). 

Researchers might usually approach this concept within various fields of study, including interpersonal relationships and organizational, cultural and motivational topics (13). 

Professionally, as different studies have shown, the hidden curriculum could be particularly effective (as an output) in medical trainees' emotional neutralization (14), changes in their ethical integrity (8), formation of their professional identity (5, 9), the professionalization process (15), students’ career choices (16), physician-patient relationship (9), students’ attitudes towards patients (17) and faculty development (18).

Since each context is unique, in many cases investigators have tried to explore and understand a hidden curriculum specific to their own academic grounds through qualitative (19 - 21) or quantitative (22) research. Despite this specificity, many extracted components of hidden curriculum are common in different studies; instances of such commonality are hierarchy (23, 24), role modeling (25, 26), haphazard interactions (8, 20), disease-centered medicine (27), etc. Considering the wide (and to some extent profound) effects of hidden curriculum, educators actively plan to approach hidden components aiming at reversing or diminishing the negative elements and bolstering or increasing the positive ones. In order to do so, they might adopt different strategies and techniques, including reflection, support systems, creative outlets, etc. to empower trainees to confront the phenomenon ‘hidden curriculum’ more effectively (28).

There has not been enough research on an accurate perception of hidden curriculum in the specific culture of Iranian health settings. Therefore, this exploratory qualitative study was conducted to determine what elements might affect Iranian medical students’ professionalism and ethical conduct at bedside through hidden curriculum.

## Method

The present study was performed to discover the specific working components of hidden curriculum regarding trainees’ medical professionalism. Medical professionalism is mostly learnt by or transferred to medical trainees under real bedside circumstances. Moreover, what is learnt by trainees in medical school could be practiced throughout their life. The emphasis and focal point of medical education at Shiraz University of Medical Sciences (SUMS) is on learning medicine through practice at bedside. For this reason, we received approval from SUMS Review Board, and consequently began an exploratory qualitative research on medical trainees between October 2014 and August 2015. 

In Iran, general medical education is a seven-year period ending in three years of clinical stages (studentship, externship and internship). Based on the study objective, the clinical phases of medical education (years 5 through 7) were important for selection of the study informants, so participants were chosen from medical students in these clinical training stages. They were recruited using the maximum variation purposive sampling technique based on sex, educational year, total average score and extracurricular activities such as active participation in social or cultural studentship affairs. All informants were confirmed to be suitable for the study based on the personal recognition of the researchers. Eventually, seventeen medical trainees including 8 females and 9 males were interviewed. 

The data were collected through semi-structured and in-depth interviews. Participants were generally briefed on the research topic and the study aims before the interview session. Interviews started with general and warm-up questions (e.g. “What do you think about the ethical climate of the educational environment at bedside?”) and continued with more specific questions (e.g. “As a student, what elements do you think are affecting your ethical/professional behavior at bedside?”). For in-depth following of the opinions and clarification of different aspects of the statements, probing questions such as the following were used: "Can you think of any examples?” "What does this mean?" and "Could you explain more?". The questions were designed based on the related literature, researchers’ personal experiences and insights achieved during interviews. The interviews generally lasted from 30 to 60 minutes. All interviews were conducted by the same person (the first author), and were audio taped and then transcribed verbatim. Data collection continued until saturation, when no new and significant data were obtained and extracted in the last two interviews.

The data were analyzed applying simple content analysis (29). The meaningful phrases (codes) were extracted from the scripts and then conceptually rearranged into the categories and themes. Disagreements in the coding process were resolved through rechecking the primary codes with the interviewees. Discussion and consensus between researchers followed in cases of disagreement over categories and themes. 

According to Guba’s criteria, there are four attributes that indicate the trustworthiness of qualitative studies: credibility, transferability, dependability and confirmability (30). In this research the following steps were taken to ensure trustworthiness: examining previous studies to design the research and plan the interviews and the primary interview questions; applying semi-structured questioning to gather data; including various informants with divergent ideas, views and experiences; endeavoring not to interfere the researchers’ beliefs and ideas in the coding and analyzing process; requesting participants to confirm the primary extracted codes of the interviews; and having the authenticity of the coding scheme reviewed and approved by two other investigators. 

*Ethical Considerations*

This study was approved by Shiraz University of Medical Sciences’ Review Board under license number 93-7264. We obtained informed consent from all participants and assured them of the confidentiality of their data at the beginning.

## Results

Seven main themes emerged from the data, revealing the factors that might affect participants in terms of professionalism and their ethical behavior. Each theme is explained through a number of sub-themes presented below:

**A) Convenient patients**

Some patients’ behavioral features and characteristics can regulate medical students' relationship with them ethically and professionally. The category consists of two sub-categories: *“patients' relationship with students” and “patients' characteristics”*.

*I. Patients’ relationship with students:*

Our findings showed that patients’ positive or negative behaviors emotionally guide medical trainees to react based on what they believe the patient deserves. According to the interviews, three main parameters that are encouraging for medical students are: "showing respect for and appreciation of trainees’ professional attempts", "patients’ trust in and compliance with students’ medical orders" and "expressing satisfaction with the trainees, especially in words". In this regard, some participants' statements are presented below: 

*“… It is a kind of self-teaching; the student himself could judge the correctness of his behavior by analyzing patients' reaction to the act. I myself can decide whether the way I behaved was appropriate or not."* [Participant No. 3]

*“There are patients that can communicate well with trainees; [as a result, medical] students try to spend more time with them since they feel they are respected by those patients.* *"* [Participant No.12]

*II. Patients’ characteristics:*

Trainees' professional thinking and conduct could be affected by a variety of other elements, such as "patients’ personal characteristics", their "socio-economic background", "type and severity of their disease" and "the state of their mental health". In this regard, some trainees stated:

*“Generally, younger patients, those with non-chronic and slight illnesses, and patients with higher levels of education and a better socio-economic position are much more respected by the medical staff. Ethical principles are also observed more closely in the case of these patients.* *"* [Participant No.10]

*“… [Once] I was in the hematology department. There were patients with specific [types of] diseases such as AIDS, hepatitis and so on. Yes, I felt the students’ behavior could be affected by these [diseases] to some extent* *"* [Participant No.12] 

Our findings implied that elements related to patients are influential in earning trainees' trust in their work. Consequently, these elements could encourage them to work with higher self-confidence and help them shift their focus from mere technical affairs to a more professional practice. 

**B) Evaluate me**

According to participants, the more seriously and precisely professionalism is evaluated as an educational competency, the more effectively trainees will try to perform in their professional function. In this regard, two sub-categories emerged according to our findings:

*I. Attention to ethics in evaluation:*

Participants believed that if responsibility, commitment, good professional relationship and similar factors were considered in evaluating the students’ competencies, they would attribute more importance to these characteristics. As a result, trainees would make an attempt to develop those competencies. In this regard, participants said:

*“Very soon [the students] might analyze the conditions surrounding the evaluation process. They might reason, “We’re not evaluated for feeling concern for the well-being of patients, so why should we care?” *[Participant No.3]

*“I never thought that our way of behaving toward patients could be important in the evaluations!* *”* [Participant No.10]

*II. Interfering factors:*

Our findings show that there are negative interfering factors that jeopardize the effectiveness of professional evaluations. For instance, the process of evaluation might easily be diverted by the interference of friendship and sexually-biased relations. This phenomenon could occur especially when the process is trusted to a senior resident without adequate higher supervisions, and will send wrong messages to medical students.

*“There isn’t a logical and legitimate [professional] evaluation. A student who is really good in terms of knowledge and professional manner may easily get lower scores than other students in view of a third person! Why? Because interpersonal relationships are at work here.* *"* [Participant No.13]

**C) Trust as the Base of Team Interactions**

Findings reveal that reciprocal relationships between medical students and the nursing team affect the quality of medical trainees' professional practice. In this regard, three sub-categories were extracted, explaining that such elements are working within medical education environments:

*I. Nurses as specific role models:*

At the bedside, medical trainees might model nurses' attitude toward patients and even try to imitate their professional practice and relationship.

*“It is working there; when [trainees] see a nurse who is really committed to observing ethical duties towards patients, they will be motivated [to be so].* *”* [Participant No. 3]

*II. Mutual understanding and positive attitude and/or behavior:*

 According to participants, the more positive and reliable the nurses were in their professional interactions with medical students, the more professionally medical trainees would interact with the staff and also with the patients. Some students confirmed this fact in their statements: 

*“I was in a ward with good-natured nurses, so I felt cheerful and could communicate easily. I was inspired by their good morale. I modeled their professional conduct and was able to learn what a professional relationship between a doctor and a nurse really means.* *"* [Participant No.14]

*“If we treat the nurses right and they behave properly toward us, tasks will be fulfilled much more effectively. Challenges among the staff could affect the morale of students negatively so they might not perform their duties as well.* *" *[Participant No.17] 

*III. Nursing ethical climate:*

Data demonstrated that cooperative interaction with the nursing team could help and encourage trainees professionally and ethically. Some instances of negative influence on students’ professional attitude towards practice include: an atmosphere of dishonesty and mistrust, a feeling of being monitored, and a feeling of duty imposed by nurses on students. In this regard, a participant explained a negative situation that is presented below:

*“[In general,] staff [nurses] influence students to a lesser degree, or their influence is temporary and transient; unprofessional or annoying behavior toward patients by a nurse or other staff might make me nervous all day, render me less effective in performing my duties, and cause me to ignore things that could increase the quality of the work.* *”* [Participant No.4]

*“Non-circular staff greatly affect the ethical climate. They’re there, it’s their ward; [therefore,] seeing the quality of their behavior toward patients and all those conventional interpersonal interactions are influential.* *"* [Participant No.12]

**D) Perceiving Encouragement**

Any perception of encouragement could help trainees be enthusiastically more active in their ethical and professional behaviors. The participants categorized the most prominent encouraging factors into the following four sub-themes:

*I. Encouragements related to patients:*

“Noticing patients regain good health” and “patients’ satisfaction with trainees' professional performance” were extracted from the data as two main sub-categories.

*“One of the encouraging factors is the patients themselves. When you solve their problem and they thank you in return…. With their eyes, by their words. This is a positive factor.* *"* [Participant No.13]

*“For instance, you may do something good for a patient, and then he or she is cooperative, makes you feel assured, or establishes a good relationship; this surely could motivate you to perform your duties better.* *"* [Participant No.17]

*II. Constructive feedback from seniors or nurses:*

According to our findings, trainees found the effect of positive encouragement rather prominent and constructive. Thus, students could be motivated when nurses or senior peers reacted to their good acts or ethical behavior with positive behavior or verbal encouragement.

*“The nursing staff could have an impression, and there may be a few [nurses] who are more extroverted. They may talk with us, tell us about their past experiences, or encourage students.* *”* [Participant No.14] 

*“[Another factor with] a great influence is peers or other trainees who are in the rotation with you. My senior trainee (a 6th year medical student) was [fortunately] supportive and encouraged me a lot.* *"* [Participant No.14] 

*III. Faculty recognition:*

Participants mentioned repeatedly that small, positive feedback on students’ behavior could be an important incentive.

*“Another positive factor is when the others understand you [and your situation]. An understanding resident or faculty member might guide you, encourage you and behave respectfully toward you, give you a kind look and criticize [something you did], and at the same time praise you.* *"* [Participant No.13]

*“When you explain something to a patient thoroughly even though it isn’t your duty, there are professors who acknowledge [the effort] and thank you [for it].* *"* [Participant No.7] 

*IV. Internal incentives:*

In addition to external incentives, there are internal ones that foster ethical and professional practice in medical students. “Having religious beliefs” and “a feeling of moral development” followed by a previous successful and prepossessing experience were among the important elements that helped trainees act as ethical agents. 

*“We can see that some internal incentives are at work here: some [students] have beliefs; religious beliefs may help, [so can] familial convictions. And personal experiences such as saving the life of a loved one could be helpful.* *”* [Participant No.13]

Therefore, according to our findings, absence of supportive and encouraging elements within the educational environment, whether internal or external, is considered a serious barrier to stability of students’ ethical behavior. In fact, receiving positive signals might guarantee students’ perseverance with good practice and ethical behavior.

**E) Relationship Satisfaction and Authenticity**

The positive attitude and professional behavior of medical students could be influenced by the timely and effective feedback that they receive from senior trainees. The participants mentioned positive reinforcement of professional behavior as an indicator/predictor of how professionally they would act and interact in their practice at the ward. Cooperative, compassionate and respectful relationships between trainees can normally enhance the quality of their professional conduct and promote ethical decision-making.

On the contrary, relationship challenges such as ridicule and blame could result in a decline in the quality of trainees' professional and ethical behavior. In this regard the participants said: 

*“It is very important that all the students in the ward be cooperative. Otherwise, challenges arise and cause a waste of energy. Surely, in a cooperative atmosphere tasks will be performed better and we will have a better attitude toward patients.* *"* [Participant No.17]

*“When together in a group, one student may be disciplined but others may not. The undisciplined ones could impose their duties on others and consequently others will take a stance against them.* *"* [Participant No.2]

**F) Workload and Students’ Well-being**

Workload and academic demands emerged in two sub-categories. Generally, students who found it difficult to see a clear rationale in the course content were also more likely to perceive their workload as being too heavy.

*I. Undesirable outcomes of substandard workload:*

In respondents’ view there was a deep relationship between workload and a number of issues related to professionalism at the bedside. The main points that participants believed were negatively affected by substandard workload included: quality of student-patient relationship, students’ ethical sensitivity, quality of undertaking professional responsibilities, students’ ability to exercise self-control in challenging situations, efficacy of medical decision-making, and having a positive attitude toward oneself. In this respect students stated:

*“In wards with higher workload, we actually see that students assume less responsibility [to perform their duties].* *”* [Participant No.4]

*“[I] really think that workload is the most important thing that makes us tired, lowers our threshold and tolerance, and subsequently causes ethics not to be practiced.* *"* [Participant No.16]

*II. Undesirable outcomes of standard situations:*

Participants believed that even when there is no real pressure resulting from substandard conditions, there might be defects related to workload such as negligence, irregularity and irresponsibility on the part of students: this might occur consciously or unconsciously. 

*“When the workload is heavy, we are too pressed for time to perform all the tasks flawlessly. However, in subsequent low workload rotations when we attend new educational wards with fewer patients and have more time, we have learned that some tasks could be done imperfectly. We might even know that we cannot do certain things without being sorry, or without being held responsible.* *"* [Participant No.11]

***G) Role Modeling at the Heart for Professionalism***

Role modeling was determined as the most important component of hidden curriculum according to our findings. Moreover, it was revealed to deeply influence the professional attitude and behavior of trainees in the settings of the research. Medical residents and particularly faculties were considered to be in the position to provide role models for participants. The importance of the issue was explained by two of the participants as follows:

*“Generally, everything in the ward depends on the faculty professor who is in charge of the monthly shifts. Everything forms based on what she or he likes or doesn’t like, and the overall atmosphere of the ward changes with that monthly change. I can say that this is true in almost all the wards that I have attended as a medical student.* *"* [Participant No.12]

*“Really, when a faculty member shows more responsibility and spends more time [with patients and on education], the effect on medical trainees will be more than hours of lecturing in the classroom.* *”* [Participant No.10]

As shown in Table 1, three categories were extracted to explain the theme through indicators and some participants’ statements. 

*I. Role modeling with regard to patients:*

Students can be professionally inspired with what they observe in role models when they treat patients or talk with them. Mostly, students would easily accept the way role models act, react or think as a mark of standard to imitate when they practice. 

*II. Regulating professionalism at the bedside:*

The data showed that role models could regulate professionalism through the positive or negative signals they send by their way of thinking and behavior towards trainees and their practice. 

*III. Non-holistic or specific role modeling*: 

This refers to the specific professional attributes of each role model and emulating the ones that acted more perfectly. According to the participants, these particularities could facilitate and increase the effectiveness of the role modeling process among medical trainees with the message to "take specific attributes from specific role models and not search for a perfect one". 

The categories, subcategories and the specific instances of each category relating to role modeling are presented in Table 1.

**Table 1:** Categories, sub-categories and participants' statements regarding the theme "Role Modeling: The Heart of Professionalism"

**Theme**	**Categories**	**Sub-Categories**	**Participants Statements**
**Role Modeling at the Heart of Professionalism**	***I.*** ***Role Modeling *** ***with Regard to *** ***Patients***	Being respectful toward patients Paying attention to patients' non- medical needs Dedicating time to patients Quality of addressing patients’ questions and concerns General sense of responsibility toward patients	*“Trainees are highly influenced by * *faculties who practice responsibly, * *have good morale, interact with * *patients respectfully, etc.” * [Participant No.17]
	***II. Regulating *** ***Professionalism *** ***at the Bedside***	Contempt for trainees' acts or attempts for any reason Discriminating against trainees on the grounds of race, gender, etc. The quality and fairness of encouragements The quality of the feedback given on trainees' practice and professional behavior Respecting students’ human dignity Emotional support in distressing situations Efficacy and accuracy of evaluation of trainees’ professional activity	*“… and when the faculty does not * *disrespect and humiliate you on the * *[educational] round [of the * *patients], even if you didn’t know the * *answer to a [scientific] question, * *and instead, concentrates on the * *teaching part, surely that affects you * *much more deeply and creates a * *mentor-mentee relationship.” * [Participant No.14]
	***III. Non-Holistic *** ***or Specific Role *** ***Modeling***	Possessing discipline, skill & professional accuracy Responsibility toward trainees Eagerness to educate Level of scientific knowledge Consistency in behaving ethically under stressful circumstances	*“I really felt like my father was * *talking to me [with all the * *compassion and responsibility]. I * *had this feeling about a few of the * *faculty professors who came to the * *ward on educational rounds ... they * *were very eager to teach students.” * [Participant No.17] *“Dr. S. gave me a deep sense of * *responsibility. That month, I learnt * *that as a physician, I should be very * *responsible toward my patients and * *their health.”* [Participant No.12]

Moreover, participants believed that picking up ethics from those with superior scientific or professional characteristics was more interesting and noteworthy than faculties with no particular attribution of morale and professionalism. In this regard one of the trainees stated: 

*“… The influence of the words of that faculty professor about ethics on students was certainly considerable as he was a high professional expert in his special field.* *"* [Participant No.5] 

## Discussion

Using an exploratory content analysis, we investigated the elements of hidden curriculum that were influential on medical students’ professional and ethical behavior during the clinical years of medical education. Consequently, seven main themes emerged as the working components of hidden curriculum specific to our context as seen in the Results section, items A to G. 

Previous studies in the field of education show that hidden curriculum consists of a spectrum of diverse components and elements (6, 31, 32). Furthermore, some authors have investigated the educational results and outcomes of hidden curriculum (15, 16, 20, 33) and the processes in which the components might work (26). The extracted components of this study can be categorized as either “human-related” or “environment-related”. Role models, patients, health staff and other trainees were the main actors of the first category while the workload, evaluation process, encouragement and supporting elements were the major components of the second one.

Findings revealed that almost all participants had experienced and were aware of the hidden components at work, but surprisingly were affected by them within the educational context. Furthermore, they believed that the impact of hidden curriculum was more than the formal trainings in several instances. Nevertheless, participants believed that either no specific plan existed, or they could not perceive that any plan was being followed for approaching influential factors. They did not have any idea for a schedule aimed at providing a guidance or management guideline for ethical and professional behaviors within educational settings. Due to this lack of general and specific plans, role models, especially faculties, undertake the above-mentioned responsibility. However, a wild fluctuation is to be expected based on faculties’ different performance or decision-making strategies. Although a great deal of positive messages could be transferred to trainees through the correct conduct of the role models, lack of practical standards might render ethical standards person-dependent and (in some instances) cause them to be neglected even though they are professionally necessary and obligatory. 

The results showed a significant part of the positive and negative effects of role models in this study were similar to other studies, whilst there were some findings dissimilar to the literature reports. Table 2 shows many characteristics of role modeling in the context of hidden curriculum, as well as characteristics mentioned both in this research and the related literature.

**Table 2:** Functioning and characteristics of role modeling based on the research data and related literature

**Common characteristics between this study and the literature**	**Extracted characteristics specific to this study**
Spending adequate time on diagnosis, treatment and educating patients (34)	Treating medical students unfairly or discriminatorily
Paying attention to the concerns of patients in addition to their medical needs (35)	Lack of discipline and precision when practicing
Feeling responsibility toward others and the medical environment (36)	Not being stable and consistent in ethical and professional behaviors
Showing humility in relationships with others (37)
Encouragement, support and/or punishment of trainees (38, 39)
Being interested in education (34)
Possessing an acceptable level of theoretical knowledge and practical skills (40)

As mentioned by the majority of participants and also in related literature, standard workload provides the condition for improving students’ ethical and professional functionality. According to the participants, standard workload could enhance the positive influences of other components of ethical hidden curriculum, and help manage and reduce the negative influences of disturbing elements. On the contrary, excessive workload is generally reported to be one of the serious impediments to the ethical and professional performance of the health staff (41, 42). Based on the findings of this study, consequences of excessive workload as one of the major problems of our educational contexts were very similar to the literature reports; nevertheless, there were a few instances that could not be found in other reports. Table 3 shows similarities and differences of the negative consequences of the workload based on the literature and our findings.

**Table 3:** Negative consequences of excessive workload based on the research data and related literature

**Common consequences between this ** **study and the literature**	**Consequences in the related ** **literature**	**Consequences extracted in ** **this study**
Stress (43 - 45)	Patient and student safety (46, 47)	Students’ moral sensitivity
Fatigue or well-being of students (42, 48, 49)	Burnout (50)	The quality of interaction with other health staff
Job satisfaction (51)	Team work (42)	
Responsibility of students (52)	Reduced inclination for working (53)	
Communication with patients and taking care of them (42)	Not spending time on personal development (53)	

According to our findings, playing an active role in the hidden curriculum, patients’ characteristics and their behaviors could affect trainees’ professional functionality either positively or negatively. This has also been shown by Higashi et al. (17) who demonstrated that medical trainees accredited the patients with labels based on their age, compliance with diagnostic and treatment plans, (not) being addicts or drug abusers, etc. Thus, medical students adjusted their professional and ethical behaviors based on these criteria. 

Participants stated that their ethical and professional behavior did not have a firm place in the process of their professional evaluation. Gaufberg et al., on the other hand, has explained in her study that students feel that they are under a sort of hidden assessment – not necessarily an official one – which actually consumes a lot of their energy (20). 

In addition to internal incentives, there are external factors that could help trainees maintain their positive performance based on the study results and also supportive literature. Rewards and punishments for specific ethical behaviors (54) and presence of supportive mechanisms for patient-centered behaviors (22) are such instances.

Contrary to our findings and to the best of our knowledge, the literature did not contain supportive data regarding the positive effects of professional interactions between nurses and medical students. As some authors have mentioned, students could help each other interpret the unwritten codes and values pertaining to clinical services (55). We also found that medical students’ professional interactions with each other, the norms associated with them and what they learned from one another were important elements of professional hidden curriculum.

Considering the seven extracted themes brings us to the conclusion that they explain the ‘ethical climate’ of our educational environment. Nevertheless, contribution of each component to the formation of such an ethical climate was different in various departments. Therefore, many students believed that ethical climates were specific to clinical departments and students usually adapt their professional and ethical behaviors to that specificity. In this way, repeating behaviors (either professional or non-professional) and adaptation to such conditions could internalize behaviors as part of trainees’ professional identity. 

Interestingly, as perceived during the study and also confirmed by a few participants, the effect of each component and the whole ethical climate on the students' professional and ethical functionality differed based on the personal characteristics of each student. In other words, trainees might be influenced based on their specific attitudes and beliefs, and the basic features of their personality. For example, a particular student might be more influenced by a professor whom she/he perceives to be more like-minded to himself/herself than other professors. In fact, we need to know that students are not passive recipients of the existing hidden curriculum, but they mostly interact actively within the context, even though they are not conscious players.

## Conclusion

Establishment of a proper professional and ethical identity in medical trainees calls for careful consideration of the influencing hidden curriculum and its working components. In this respect, medical trainees' related attitudes, beliefs and experiences were investigated as a valuable source for recognizing and exploring the native condition of Iranian educational health settings.

In this regard, seven main themes including human-related and environment-related elements were extracted as components of the ethical hidden curriculum. Role modeling, health staff, peer students, patients, workload, systematic evaluation of ethics and professionalism, and encouraging incentives were the main players of the ethical hidden curriculum. All components were important in the formation of the current 'ethical climate' while, according to the participants, the weight of each component differed according to the department. Moreover, the effect of the ethical climate on each trainee was largely dependent on his/her personal characteristics, attitudes and beliefs. 

Exploring the working hidden curriculum components in particular settings helps devise better plans to direct ethical and professional behaviors. Here are some suggestions for improving professionalism and the ethical conduct of medical students through hidden curriculum based on the findings of the study:

- Raising consciousness among faculties and educators about the importance and impression of hidden curriculum beside formal education as well as their active role in improving medical students’ professionalism through it
- Improving evaluation processes regarding professionalism and students’ ethical manner
- Improvement of workload through implementation of standards and improving duty hours while maintaining the quality of education
- Empowering students to deal with hidden curriculum actively, to select positive messages and to manage and control negative ones
- Encouragement of students’ ethical acts through systematic support, and systemic handling of discouraging factors and elements
